# Characterization of Hair Cell-Like Cells Converted From Supporting Cells After Notch Inhibition in Cultures of the Organ of Corti From Neonatal Gerbils

**DOI:** 10.3389/fncel.2018.00073

**Published:** 2018-03-20

**Authors:** Yi Li, Shuping Jia, Huizhan Liu, Tomoko Tateya, Weiwei Guo, Shiming Yang, Kirk W. Beisel, David Z. Z. He

**Affiliations:** ^1^Department of Otorhinolaryngology, Beijing Tongren Hospital, Beijing Capital Medical University, Beijing, China; ^2^Department of Biomedical Sciences, Creighton University School of Medicine, Omaha, NE, United States; ^3^Institute for Virus Research, Kyoto University, Kyoto, Japan; ^4^Department of Otorhinolaryngology, PLA General Hospital, Beijing, China

**Keywords:** notch signaling, hair cells, stereocilia formation, mechanotransduction, development, hearing loss, supporting cells, prestin

## Abstract

The senses of hearing and balance depend upon hair cells, the sensory receptors of the inner ear. Hair cells transduce mechanical stimuli into electrical activity. Loss of hair cells as a result of aging or exposure to noise and ototoxic drugs is the major cause of noncongenital hearing and balance deficits. In the ear of non-mammals, lost hair cells can spontaneously be replaced by production of new hair cells from conversion of supporting cells. Although supporting cells in adult mammals have lost that capability, neonatal supporting cells are able to convert to hair cells after inhibition of Notch signaling. We questioned whether Notch inhibition is sufficient to convert supporting cells to functional hair cells using electrophysiology and electron microscopy. We showed that pharmacological inhibition of the canonical Notch pathway in the cultured organ of Corti prepared from neonatal gerbils induced stereocilia formation in supporting cells (defined as hair cell-like cells or HCLCs) and supernumerary stereocilia in hair cells. The newly emerged stereocilia bundles of HCLCs were functional, i.e., able to respond to mechanical stimulation with mechanotransduction (MET) current. Transmission electron microscopy (TEM) showed that HCLCs converted from pillar cells maintained the pillar cell shape and that subsurface cisternae, normally observed underneath the cytoskeleton in outer hair cells (OHCs), was not present in Deiters’ cells-derived HCLCs. Voltage-clamp recordings showed that whole-cell currents from Deiters’ cells-derived HCLCs retained the same kinetics and magnitude seen in normal Deiters’ cells and that nonlinear capacitance (NLC), an electrical hallmark of OHC electromotility, was not detected from any HCLCs measured. Taken together, these results suggest that while Notch inhibition is sufficient for promoting stereocilia bundle formation, it is insufficient to convert neonatal supporting cells to mature hair cells. The fact that Notch inhibition led to stereocilia formation in supporting cells and supernumerary stereocilia in existing hair cells appears to suggest that Notch signaling may regulate stereocilia formation and stability during development.

## Introduction

The mammalian auditory sensory epithelium, the organ of Corti, contains hair cells and supporting cells. Hair cells, with the hallmark of the stereocilia bundle in their apical surface, transduce mechanical stimuli into electrical activity. Mechanoelectrical transduction is mediated by the hair bundle, an array of modified microvilli or stereocilia arranged in a staircase on the apical surface of the hair cell (Fettiplace and Hackney, 2006). In addition to the stereocilia bundle, Hair cells also contain structural and functional specializations in the basolateral and synaptic membranes, which are responsible for electrical and mechanical activities and synaptic transmission. The supporting cells, which include pillar cells, Deiters’ cells, and inner phalangeal cells contribute to the stiffness of the cochlear partition and the homeostasis of the ionic and chemical environment (Slepecky, 1996; Raphael and Altschuler, 2003).

Loss of hair cells as a result of aging or exposure to noise and ototoxic drugs is the major cause of noncongenital hearing and balance deficits. Studies in the last three decades have demonstrated that hair cells in non-mammals can spontaneously be replaced by production of new hair cells through transdifferentiation and proliferation of supporting cells. Although supporting cells in adult mammals have lost the capability to spontaneously proliferate and transdifferentiate, neonatal and adult supporting cells are able to convert to hair cells after overexpression of *Atoh1* or inhibition of Notch signaling (Zheng and Gao, 2000; Zheng J. L. et al., 2000; Zine et al., 2000; Kawamoto et al., 2003; Izumikawa et al., 2005; Kelly et al., 2012; Mizutari et al., 2013). The Notch signaling pathway, an evolutionary conserved molecular mechanism involved in the determination of a variety of cell fates (Andersson et al., 2011), plays multiple roles during vertebrate inner ear morphogenesis (Kelley, 2006; Kiernan, 2013). Notch signaling first specifies prosensory progenitors through lateral induction (Kiernan et al., 2006; Hartman et al., 2010) and subsequently directs progenitors to further differentiate into supporting cells by preventing supporting cells from adopting hair cell fate through lateral inhibition (Adam et al., 1998; Daudet and Lewis, 2005). Since Lanford et al. (1999) first showed that a decrease in Notch activation by genetic deletion of *Jag2*, the ligand of Notch1 receptor, resulted in a significant increase in sensory hair cells, numerous *in vivo* and *in vitro* studies have confirmed that inhibition of Notch signaling by genetically engineered mutations or pharmacological inhibition generates ectopic hair cells (Zine et al., 2000; Yamamoto et al., 2006; Pan et al., 2010; Zhao et al., 2011; Kelly et al., 2012; Bramhall et al., 2014; Basch et al., 2016; Maass et al., 2016). These hair cells, located in the regions of phalangeal cells, pillar and Deiters’ cells, express Myo7a (a hair cell marker) and have stereocilia bundle on their apical surface. However, it is unclear whether these cells possess the structural and physiological properties similar to hair cells or the supporting cells from which they are derived. Studies using immunocytochemistry suggest that they remain immature hair cells (Kelly et al., 2012; Liu et al., 2012). In the present study, we used electron microscopy and cellular electrophysiological techniques to examine stereocilia, mechanotransduction (MET), basolateral membrane structure, and electrophysiological properties of ectopic hair cells (defined hair cell-like cells or HCLCs) in cultures of the organ of Corti from neonatal gerbils after pharmacological inhibition of Notch signaling. Characterizing properties of these HCLCs addresses the question of whether inhibition of Notch signaling after birth is sufficient to convert supporting cells to hair cells that can transduce mechanical stimulation and function as a mature hair cell. Therefore, this study will provide important information for future studies that use Notch inhibition strategy to convert supporting cells to hair cells to restore hearing.

## Experimental Procedures

### Tissue Culture of the Organ of Corti

Care and use of the animals in this study were approved by the Institutional Animal Care and Use Committees of Creighton University and Beijing Capital Medical University. The methods were carried out in accordance with the approved guidelines.

Newborn Mongolian gerbils (*Meriones unguiculatus*) were used for all experiments. Births in the gerbil breeding colonies were monitored at 9:00 A.M. and 5:00 P.M. daily. Cochlear explantation was performed on postnatal day 0 (P0) or P5. A detailed description has been given previously (He, 1997; Jia et al., 2009). In brief, newborn gerbil pups were given a lethal dose of sodium pentobarbital (200 mg/kg, i.p.). The animals were decapitated and the cochleae were dissected out and kept in L-15 medium (Life Technologies, Grand Island, NY, USA). The basilar membrane and the associated organ of Corti were unwrapped from the modiolus. The apical and basal turns were dissected out and explanted onto 5 × 5 mm plastic coverslips placed on the bottom of culture dishes containing 1.2 ml of Dulbecco’s Modified Eagle Medium (DMEM, Life Technologies) with high glucose. The tissue was oriented with the ciliated pole of hair cells pointing upward. Culture medium contained 10 percent (by volume) of heat-inactivated fetal bovine serum, 2% of B-27, and 1% of N-2 supplements (all from Invitrogen). Five micromolar DAPT or LY-411575 (Sigma-Aldrich, St. Louis, MO, USA), dissolved in 0.02% of DMSO, were also added to the dish (Yamamoto et al., 2006). The cultures were maintained in a 37°C incubator with 5% CO_2_. After 48 h, the culture was washed and replaced with normal culture medium containing no DAPT or LY-411575. In control cultures, only an equal amount (0.02%) of DMSO was included in the culture medium. The culture medium in all cultures was replaced every other day. All data described in this study came from cultures (prepared from P0 or P5 gerbils) treated with 5 μM DAPT or LY-411575 for 48 h followed by in normal culture medium (condition) for 6–10 additional days.

### Recording Mechanotransduction (MET) Current and Nonlinear Capacitance (NLC)

The culture was bathed in L-15 medium in an experimental chamber mounted on the stage of an upright Leica microscope with Burleigh platform. Recording MET current from cultured Outer hair cells (OHCs) was essentially the same as recording from freshly isolated cochlear segments (Jia et al., 2009). The inorganic components (in mM) of L-15 medium were as follows: NaCl, 136; NaH_2_PO_4_, 5.8; KCl, 5.4; CaCl_2_, 1.3; MgCl_2_, 0.9; and MgSO_4_, 0.4. The solution was buffered with 10 mM HEPES, the pH was adjusted to 7.4, and osmolarity was 300 mOsm. The patch electrodes were backfilled with an internal solution, which contained the following (in mM): CsCl (or KCl for whole cell current recording), 140; CaCl_2_, 0.1; MgCl_2_, 3.5; MgATP, 2.5; EGTA-KOH, 5; and HEPES-KOH, 10. The solution was adjusted to pH 7.4 with Trizma Base and osmolarity adjusted to 300 mOsm with glucose. The pipettes had initial bath resistances of ~2–4 MΩ. Access resistances were typically from 6 to 10 MΩ. After the whole-cell configuration was established and series resistance was ~70% compensated, the cell was held under voltage-clamp mode to record MET currents in response to bundle deflection by a fluid jet (with pipette tip diameter of ~10 μm) positioned ~10–15 μm away from the bundle. Sinusoidal burst (100 Hz) was used to drive the fluid jet (Kros et al., 1992; Jia et al., 2009). The magnitude of the bundle motion during experiments was set to 350 nm, sufficient to evoke saturated MET currents while avoiding damage to the tip-link. The holding potential was set at −70 mV during recording. The currents, filtered at 2 kHz, were amplified using an Axopatch 200B amplifier. Currents were acquired by software pClamp 9.1 running on an IBM-compatible computer with a 16-bit analog-to-digital converter (Digidata 1322B). The sampling frequency was 20 kHz. Three presentations were averaged for each trial. Data were analyzed using Clampfit in the pClamp software package and Igor Pro (WaveMetrics, Inc., Portland, OR, USA).

For nonlinear capacitance (NLC) measurements, the culture was bathed in extracellular solution containing the following (in mM): NaCl, 120; TEA-Cl, 20; CoCl_2_, 2; MgCl_2_, 2; HEPES, 10; and glucose, 5 at pH 7.4. The internal solution contained the following (in mM): CsCl, 140; MgCl_2_, 2; EGTA, 10; and HEPES, 10 at pH 7.4. The two-sine voltage stimulus protocol (10 mV peak at both 390.6 and 781.2 Hz) with subsequent fast Fourier transform-based admittance analysis was used to measure membrane capacitance (Wang et al., 2010). NLC data were acquired using jClamp software^1^. Fits to the capacitance data were made in IgorPro. The NLC can be described as the first derivative of a two-state Boltzmann function relating nonlinear charge movement to voltage (Santos-Sacchi, 1989). The capacitance function is described as:

(1) $$C_{\text{m}}\;=\;\frac{Q_{\max}\alpha }{\exp[\alpha (V_{\text{m}}-V_{1/2})]{\left(1+\exp[-\alpha (V_{\text{m}}-V_{1/2})]\right)}^{2}}+C_{\text{lin}}$$

In equation 1, *Q*_max_ is maximum charge transfer; *V*_1/2_ is the voltage at which the maximum charge is equally distributed across the membrane, or equivalently, the peak of the voltage-dependent capacitance; *C*_lin_ is linear capacitance; and *α* = *ze/kT* is the slope of the voltage dependence of charge transfer where *k* is Boltzmann’s constant, *T* is absolute temperature, *z* is valence of charge movement, and *e* is electron charge.

### Immunocytochemistry and Electron Microscopy

Cultures were perfused with 2% formaldehyde in phosphate buffer. After 15 min fixation, the tissues were treated with 0.2% Triton X-100/PBS. Goat serum (10%) was used to block nonspecific binding. The tissue was then incubated with anti-Myo7a antibodies (Product #: 25-6790, Porteus BioSciences, Inc., Ramona, CA, USA). The samples were washed with PBS, followed by incubation with secondary antibodies. After being washed with PBS, the samples were mounted on glass slides with antifade solution (Prolong Antifade Kit, Life Technologies, Grand Island, NY, USA). To visualize the stereocilia, actin in stereocilia was labeled with rhodamine-phalloidin (Life Technologies), as described previously (Yang et al., 2012). The samples were examined using a LSM 510 META confocal scanning system with three lasers mounted on a Zeiss AxioPlan 2IE MOT motorized upright microscope.

For scanning electron microscopy (SEM), the organ cultures were fixed for 15 min with 2.5% glutaraldehyde in 0.1 M sodium cacodylate buffer, pH 7.4, containing 2 mM CaCl_2_, washed in PBS, and then postfixed for 10 min with 1% OsO_4_ in the same buffer and washed. The tissues were dehydrated via an ethanol series, critical point-dried from CO_2_ and sputter-coated with gold. The tissue was then examined using an FEI Quanta 200 scanning electron microscope.

For transmission electron microscopy (TEM), the organ cultures were fixed with 2.5% glutaraldehyde, washed in PBS, and then postfixed for 10 min with 1% OsO_4_. After being washed, the cultures were dehydrated in acetone and embedded in a mixture of Araldite and Epon 812 in flat rubber molds. The blocks were then mounted in a Leica Ultracut. Two micrometer semi-thin sections were cut with a glass knife and stained with 1% Toluidine blue in 0.5% borax buffer. When a location of interest was found, 60-nm thin sections were cut with a diamond knife and collected on 300-mesh grids. After being stained with 3% uranyl acetate for 15 min and 1.5% lead citrate for 3 min, the samples were examined in an FEI Tecnai G2 Spirit transmission electron microscope and photographed.

### Statistical Analysis

Means and standard deviations were calculated based on measurements from different samples and biological repeats. Student’s *t*-test was used to determine statistical significance between two different conditions (such as DAPT-treated and control tissues) and developmental stages. Probability (*P*) value ≤ 0.01 was regarded as significant.

## Results

### Generation of Stereocilia Bundles in Supporting Cells

**Figure 1:**
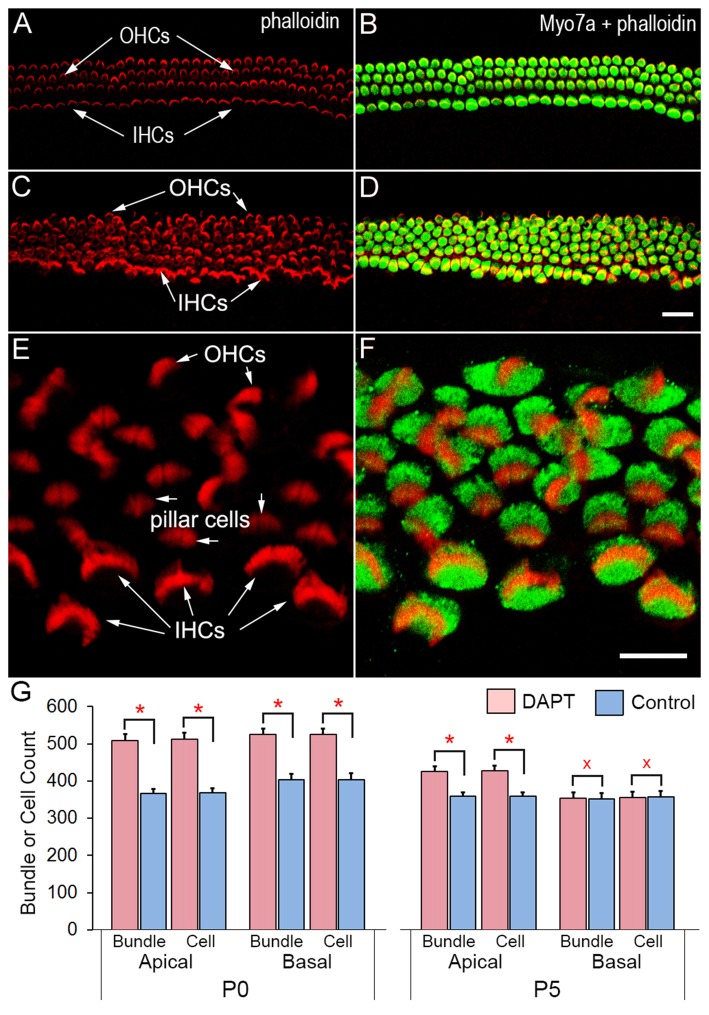
Confocal images of stereocilia bundles and hair cells in cochlear explants of neonatal gerbils. The stereocilia bundle was labeled with rhodamine-phalloidin and soma was labeled with anti-Myo7a antibodies. **(A,B)** Images obtained from control cultures. **(C,D)** Images from DAPT-treated cultures. Bar in **(D)**: 10 μm for **(A–D)**. **(E,F)** Images of a DAPT-treated culture under high magnification. Bar in **(F)**: 10 μm for **(E,F)**. **(G)** Count of stereocilia bundles from cultures prepared from P0 and P5 gerbil cochleae. Two regions (1 mm in length in each region) in the apical and basal turns were examined. Statistical significance (marked by red asterisks with *p* ≤ 0.01 regarded as significant, *n* = 3, student’s *t*-test) was found between DAPT-treated and control groups. No statistical significance (*p* ≥ 0.05, *n* = 3) was found between control and DAPT-treated cultures prepared from the basal turn of P5 gerbils (marked by red Xs).

Hair cells and supporting cells in the organ of Corti are morphologically differentiated at birth, although they undergo further development after birth (He et al., 1994; Souter et al., 1997; Kros et al., 1998; Waguespack et al., 2007; Lelli et al., 2009). We examined whether inhibition of Notch signaling could induce stereocilia bundle genesis in supporting cells. Organotypic cultures of the organ of Corti from P0 gerbils were prepared for the experiments. The onset of hearing occurs between P10 and P12 in gerbils (Woolf and Ryan, 1984), similar to other altricial rodents such as mouse, rat, and hamster. We chose gerbils because their hair cells and stereocilia bundles are larger than those of mice, which is advantageous to electrophysiological recording and mechanical stimulation. Furthermore, information regarding morphological and functional development of hair cells, especially ultrastructure of hair cells, is available for reference in this species (He et al., 1994; Weaver and Schweitzer, 1994; Souter et al., 1997). Since the activation of Notch pathways requires γ-secretase activity (De Strooper et al., 1999), we blocked Notch activity by adding 10 μM DAPT, a γ-secretase inhibitor, in the culture medium for 48 h. Figure 1 shows some representative confocal images of stereocilia bundles and hair cells in DAPT-treated (i.e., 2-day DAPT treatment, followed by 6 days in normal culture medium) and control (with 0.02% of DMSO for 2 days) cochlear explants after 8 days in culture. We labeled the stereocilia bundles with rhodamine-phalloidin and the soma with anti-Myo7a antibodies. As shown, the control exhibits three rows of well-organized stereocilia bundles of OHCs and one row of inner hair cells (IHCs; Figures 1A,B). In contrast, 5–6 rows of hair bundles with Myo7a-positive soma are seen in the DAPT-treated culture (Figures 1C,D). The stereocilia bundles in the DAPT-treated culture exhibit different orientations, sizes and shapes (Figures 1E,F). To determine how many new stereocilia bundles were generated, we counted the total number of stereocilia bundles and Myo7a-positive cells in two regions (1 mm in length in each region) in the apical and basal turns from DAPT-treated and control cultures (three cultures for each group) using off-line confocal images. The total bundle and cell counts are presented in Figure 1G. As shown, there are significant increases in the number of stereocilia bundles and Myo7a-positive cells in both regions. The increase, which has been reported in previous studies (Yamamoto et al., 2006; Zhao et al., 2011), is due to conversion of supporting cells to HCLCs. We also examined the number of stereocilia bundles and Myo7a-positive cells from cultures prepared from P5 gerbils using the same DAPT-treatment scheme as for the P0 cultures (i.e., 2-day DAPT treatment, followed by 6 days in normal culture medium). As shown in the right panel of Figure 1G, the increase in the number of stereocilia bundles in the apical turn region is not as dramatic as that seen in cultures prepared from P0 gerbils. In the basal turn, no significant difference in bundle or cell count is seen between DAPT-treated and control groups. This age-dependent effect of Notch inhibition was reported before (Yamamoto et al., 2006; Zhao et al., 2011; Liu et al., 2012). Our result is consistent with those studies.

**Figure 2:**
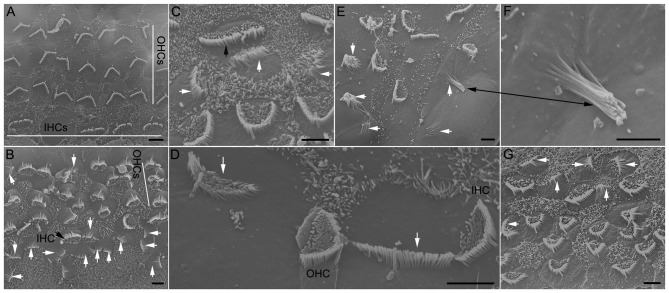
Scanning electron microscopy (SEM) micrographs of stereocilia bundles from 8-day-old cultures prepared from the low apical turn. **(A)** An SEM micrograph obtained from a culture without DAPT treatment (control). Inner hair cell (IHC) and outer hair cell (OHC) regions are marked by white lines. **(B–F)** Examples of stereocilia bundles of hair cell-like cells (HCLCs) from DAPT-treated cultures. A black arrow in **(B)** or **(C)** indicates the stereocilia bundles of IHCs. White arrows in **(B–E)** indicate various forms of stereocilia bundles seen in the apical surface of HCLCs. Black arrows in **(E)** and **(F)** mark the same stereocilia bundle with a higher magnification in **(F)**. **(G)** SEM image from a culture treated with 5 μM LY411575. Scale bars: 5 μm for all images.

Although the increase in the number of stereocilia bundles and Myo7a-positive cells as a result of Notch inhibition has been shown by numerous studies, few studies have examined ultrastructure of stereocilia bundles using SEM and TEM. We used SEM to examine stereocilia bundle morphology in DAPT-treated and control cultures (all prepared from P0) after 8 days *in vitro*. As shown in Figure 2, several differences are apparent between the two groups: First, in control cultures there are four rows of stereocilia bundles although the alignment of three row of OHCs and one row of IHCs was disrupted due to the growth and shift of different tissue constituents in culture (Figure 2A). No stereocilia bundles are seen in the apical surface of supporting cells. In DAPT-treated cultures, however, 5–6 rows of stereocilia bundles are seen (Figure 2B). Stereocilia bundles are present in the apex of not only hair cells but also supporting cells (marked by white arrows in Figures 2B–G). Second, stereocilia in control cultures display well-organized bundle morphology with kinocilium centered in the “V”-shaped stereocilia (Figure 2A). In contrast, newly emerged stereocilia in the apex of HCLCs display bundle formation with various size, shape and length. While some newly emerged bundles appear to have 2–4 rows of stereocilia organized in a staircase fashion with a kinocilium in the middle (marked by a white arrow in Figure 2D), most bundles display no clear sign of staircase pattern (marked by white arrows in Figures 2C–F). Some bundles have the shape and organization resembling vestibular hair cells (marked by black arrows in Figures 2E,F). Furthermore, stereocilia seen in HCLCs appear to be thinner than those seen in endogenous hair cells, as shown in Figure 2C (black arrow marks an IHC stereocilia bundle, while white arrows mark stereocilia of three HCLCs). Third, microvilli appear to be more abundant in DAPT-treated cultures (Figure 2B) than in control cultures (Figure 2A). It is interesting to note that, while microvilli in DAPT-treated cultures are abundant in endogenous hair cells, most HCLCs no longer exhibit microvilli (Figures 2C–F), suggesting that microvilli may have been used to form the stereocilia bundle in those cells. This is consistent with the notion that microvilli may form the building block for stereocilia (Gorelik et al., 2003).

We also used a different substitute for DAPT just to rule out the possibility that stereocilia formation in supporting cells was due to effort of DAPT that may be unrelated with Notch inhibition. LY411575 is also a γ-secretase inhibitor that was shown to block Notch activation *in vivo* and *in vitro* (Mizutari et al., 2013; Bramhall et al., 2014). Treatment of the cultures with LY411575 also resulted in stereocilia bundle formation in supporting cells (Figure 2G, marked by white arrows), thus, paralleling the results observed in DAPT-treated cultures.

**Figure 3:**
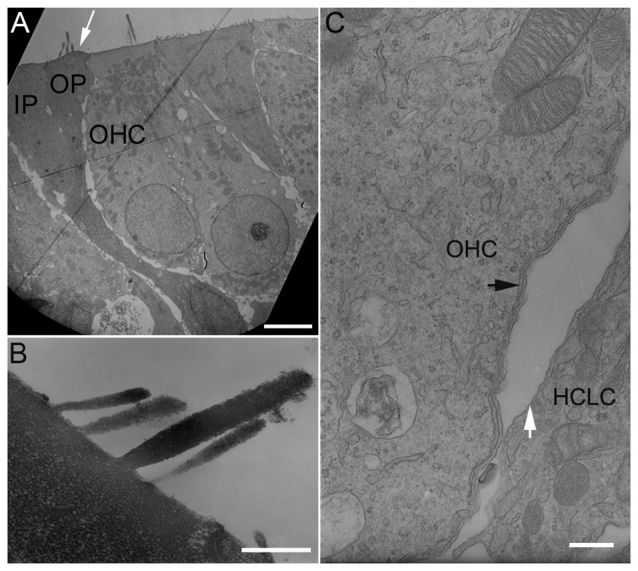
Transmission electron microscopy (TEM) micrographs of hair cells and HCLCs. **(A)** Hair cells and a stereocilia bundle-bearing pillar cell. IP: Inner pillar cell. OP: Outer pillar cell. OHC: Outer hair cell. Bar: 5 μm. **(B)** Stereocilia of an HCLC. Bar: 1 μm. **(C)** Lateral walls of an OHC and an adjacent HCLC. Black arrows indicate one layer of subsurface cisternae underneath the plasma membrane of the OHC. A white arrow indicates the plasma membrane of an adjacent HCLC. No subsurface cisternae were seen. Bar: 0.2 μm.

We examined stereocilia bundle morphology of HCLCs using TEM. As shown in Figure 3A, the stereocilia bundle (marked by a white arrow) is clearly visible on the apical surface of an outer pillar cell (OP) located between an OHC and an inner pillar cell (IP). Figure 3B exhibits the ultrastructure of the stereocilia from an HCLC under high magnification. As shown, the ultrastructure of the HCLC stereocilia resembles that of hair cells.

The OHC lateral wall is a unique trilaminate structure consisting of the plasma membrane, the cortical lattice, and subsurface cisternae (He et al., 2010). In altricial rodents such as mouse, rat and gerbil, subsurface cisternae develop after birth and the first layer occurs between P6 and P7 (Weaver and Schweitzer, 1994; Souter et al., 1997). TEM was used to examine the ultrastructure of the lateral wall of HCLCs in the OHC region from three cultures. Figure 3C shows a TEM micrograph of the lateral walls of an HCLC and an adjacent OHC from a basal turn culture 8 days after DAPT treatment. As shown, one layer of subsurface cisternae (marked by a black arrow in Figure 3C) is already present beneath the plasma membrane in the OHC. In contrast, the adjacent HCLC (marked by a white arrow) shows no sign of subsurface cisternae.

### Supernumerary Stereocilia in Endogenous Hair Cells

**Figure 4:**
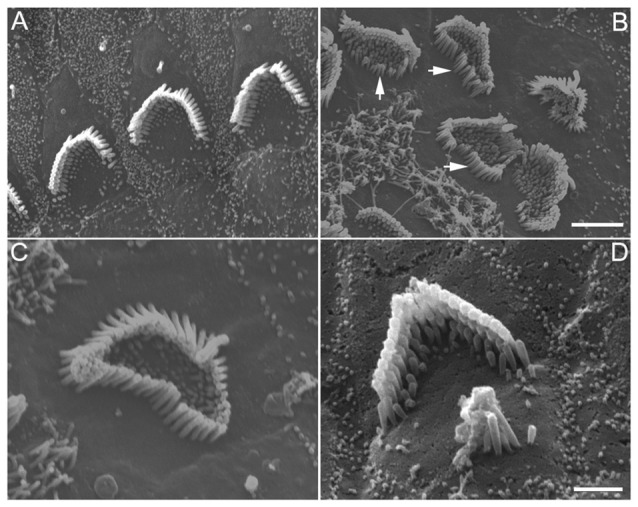
SEM images of stereocilia bundles of hair cells from 8-day-old cultures prepared from the low apical or middle turn. **(A)** Hair cells without DAPT treatment. **(B–D)** Hair cells treated with 5 μM DAPT. Various forms of supernumerary stereocilia were observed in existing hair cells after Notch inhibition. Scale bars: 5 μm. Bar in **(B)** also applies to **(A)**, while bar in **(C)** also applies to **(D)**.

The stereocilia bundle morphology of endogenous hair cells was examined using SEM. Some representative SEM images obtained from control and DAPT-treated cultures (from low apical turn) are presented in Figure 4. As shown in Figure 4A, stereocilia of endogenous hair cells maintain their shape, length and stair-case organization with some remaining microvilli in control cultures. In DAPT-treated cultures, however, signs of supernumerary stereocilia were apparent, despite the fact that the stereocilia of hair cells still maintained their length and stair-case organization. The signs of supernumerary stereocilia included an increase in the number of stereocilia (Figures 4B,C) and/or emergence of an extra stereocilia bundle next to the existing one (Figure 4D). We counted the total number of stereocilia from nine hair cells from the low apical turn from DAPT-treated and control cultures. The number of stereocilia was 95.3 ± 11.5 and 67.2 ± 7.8 for hair cells in DAPT-treated and control cultures, respectively. Thus, a significant increase (*p* ≤ 0.01) in the total number of stereocilia was seen after Notch inhibition. The supernumerary stereocilia appeared to be from elongation of microvilli, as evident in Figure 4B (with white arrows indicating elongating microvilli). In addition to supernumerary stereocilia, microvilli in most hair cells were still abundantly present (Figure 4B). This is in contrast to control cultures where most microvilli in hair cells were absorbed after 8 days in culture. Interestingly, while Notch inhibition led to supernumerary stereocilia in endogenous hair cells, the length of stereocilia bundles did not increase.

### Mechanotransduction of Newly Emerged Stereocilia Bundles

**Figure 5:**
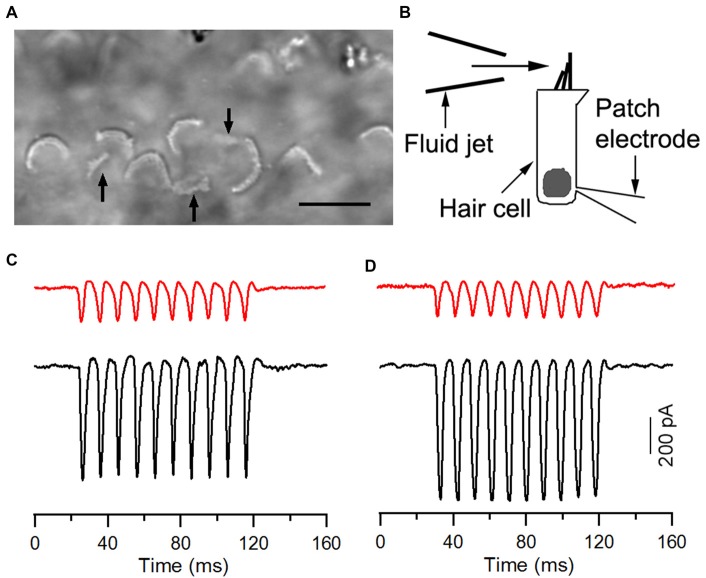
Mechanotransduction (MET) currents recorded from ectopic hair cells. **(A)** Image of stereocilia bundles under bright field illumination with a water-immersion objective (63×, NA: 0.85). Arrows indicate ectopic bundles (from HCLCs), which appeared smaller in size with no obvious “V” shape. We avoided recording from any cells when their identity was ambiguous. **(B)** Schematic drawing of recording MET from cultured hair cells using the whole-cell voltage-clamp technique. The bundle was stimulated with the fluid jet. **(C)** Representative MET currents recorded from an HCLC (red) and an IHC (black) in the IHC region. **(D)** MET currents recorded from an HCLC (red) and an OHC (black) in the OHC region. Recordings were made from 8-day-old cultures.

We measured the MET current from HCLCs in the IHC and OHC regions to determine whether HCLCs were able to transduce mechanical stimulation to electrical response. HCLCs in cultures could easily be identified with a 63× water immersion objective (NA: 0.85) when displayed in a TV monitor after additional optical and electronic magnification. The hair bundles behaved as light pipes and appeared as bright V- or curved-shaped lines under bright-field illumination (Ricci et al., 2000; Jia and He, 2005). Identification of HCLCs was based on their bundle size and shape. Most of their bundles were not in “V”-shape and orientated in different directions. The bundles appeared to be smaller in size when compared to those of hair cells. Furthermore, HCLCs were more often found medial to IHCs, or between IHCs and OHCs. Some examples of stereocilia bundles of HCLCs (marked by black arrows) from light microscopy are presented in Figure 5A. We only chose those cells that had clear signs of the bundle characteristics described above and avoided recording from any cells whose identity was ambiguous. The bundle was deflected with the fluid jet technique (Kros et al., 1992; Jia et al., 2009) and deflection-evoked MET was recorded (Figure 5B). MET currents from adjacent hair cells were also recorded for reference. Deflection of the bundle evokes large MET currents from IHCs and OHCs, as shown in Figures 5C,D. The current was asymmetrical, with inward current being greater than the outward current (black traces in Figures 5C,D). We measured maximal MET currents from eight IHCs and ten OHCs from three cultures. The mean magnitude of the current was 411 ± 35 and 626 ± 94 pA, respectively, comparable to the magnitude observed in rodent hair cells (Kennedy et al., 2003; Waguespack et al., 2007). We also measured MET currents from 19 and 12 HCLCs adjacent to IHCs and OHCs, respectively, from the same cultures. Fifteen (79%) and nine (75%) cells responded positively to the mechanical stimulation. As shown in Figures 5B,C (red traces), the MET current from HCLCs was also highly asymmetrical, resembling that of hair cells. The mean magnitude of the maximal current was 216 ± 59 and 201 ± 58 pA, respectively. The presence of MET currents from HCLCs suggests that newly emerged stereocilia bundles are functional despite the fact that the magnitude of the MET current was less than that of hair cells.

### Expression of Outward Currents and Prestin in HCLCs

**Figure 6:**
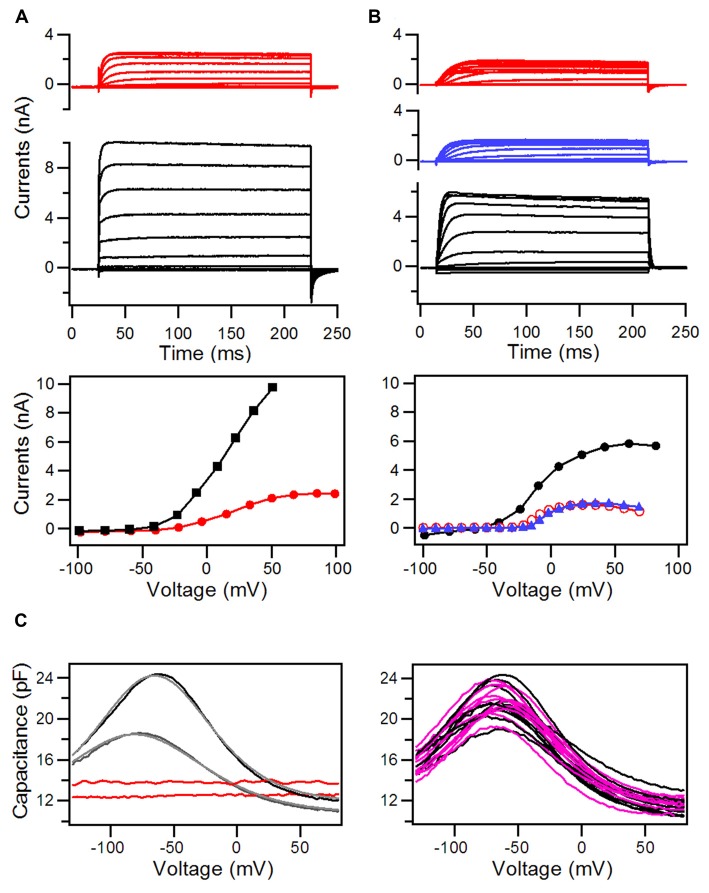
Whole-cell currents and nonlinear capacitance (NLC) recorded from hair cells and HCLCs in the regions of IHCs and OHCs from 8-day-old cultures with DAPT treatment. **(A)** Whole-cell currents recorded from an HCLC (red) and an IHC (black) in the IHC region. I-V curves from the steady-state response of the whole-cell current are plotted in the middle panel. **(B)** Whole-cell currents recorded from an HCLC (red) and an OHC (black) in the OHC region. I-V curves are plotted in the middle panel. **(C)** NLC measured from HCLCs in cultures at 8 and 12 days. NLC from recorded from OHCs in the same preparation was also shown for comparison.

In addition to apical specialization that is responsible for MET, hair cells also have specializations in the basolateral membrane that are responsible for electrical and mechanical properties of hair cells. Using voltage-clamp technique, we first measured whole-cell currents from HCLCs in the IHC and OHC regions to determine whether they have similar ion channel kinetics as those observed in IHCs and OHCs. We compared the magnitude and kinetics of the whole cell currents at 8 days after DAPT treatment. Figure 6A shows the whole-cell currents obtained from an IHC (black traces) and an HCLC (red traces) in the IHC region, whereas Figure 6B exhibits the currents from an OHC (black traces) and an HCLC (red traces) in the OHC region. As shown, the outward K^+^ currents in hair cells and HCLCs have different magnitudes and activation kinetics. The outward currents seen in HCLCs resembled those of Deiters’ cells (Nenov et al., 1998).

Prestin-based somatic motility is a unique property of OHCs (Zheng J. et al., 2000). Although OHC electromotility is first detected at P6 in developing gerbils and in cultures (He et al., 1994; He, 1997), NLC, an electrical signature of motility (Ashmore, 1989; Santos-Sacchi, 1989), can be detected as early as P3 (Oliver and Fakler, 1999; Belyantseva et al., 2000). The magnitude of NLC increases until adult-like response is reached at P14 (Oliver and Fakler, 1999). We measured NLC from HCLCs and adjacent OHCs at two different time points (8 and 12 days after DAPT treatment). OHCs in the same region were used as controls. Figure 6C shows some examples of those measurements. As shown, the OHC displays a robust bell-shaped NLC at 8 days in culture. The NLC response became significantly larger at 12 days in culture. In contrast, HCLCs showed a flat response with no signs of any bell-shaped voltage dependency at 8 or 12 days.

## Discussion

Although ample studies have demonstrated that inhibition of Notch signaling can lead to the production of HCLCs from transdifferentiation of supporting cells in the organ of Corti from neonatal animals (Zine et al., 2000; Yamamoto et al., 2006; Pan et al., 2010; Zhao et al., 2011; Kelly et al., 2012; Bramhall et al., 2014; Basch et al., 2016; Maass et al., 2016), no studies have systematically examined morphological and electrophysiological properties of these cells. A few studies have examined MET of HCLCs using FM1–43 dye and the expression of prestin with anti-prestin antibodies. The results are inconclusive as FM1–43 dye can also get into the cells by the process of endocytosis (Griesinger et al., 2002, 2004) and different nonselective ion channels (Meyers et al., 2003). Conclusions from prestin-antibody-based immunocytochemistry are inconsistent among different studies (Kelly et al., 2012; Liu et al., 2012, 2014; Mizutari et al., 2013; Bramhall et al., 2014). In the present study we used electron microscopy and cellular electrophysiological techniques to examine morphological and electrophysiological features that reflect important hair cell properties such as MET and membrane conductances. We also examined unique features (such as NLC and subsurface cisternae) that represent the basolateral membrane property of OHCs. These studies allowed us to unequivocally determine whether HCLCs possess some properties that mature hair cells should normally have.

We showed that reduction of Notch activity led to generation of stereocilia in HCLCs (Figure 3). The stereocilia appear to be originated from elongation of microvilli as microvilli were abundant on top of nascent supporting cells after birth and disappearance of microvilli appeared to be coincident with diminishing capability of supporting cells to transdifferentiate to hair cells (Figure 1G). Furthermore, most HCLCs no longer exhibited microvilli (Figures 2C–F), suggesting that microvilli may have been used to form stereocilia bundles in those cells. This supports the notion that microvilli may provide the building block for stereocilia (Gorelik et al., 2003). SEM examination showed that some bundles of HCLCs had 2–4 rows of stereocilia organized in a staircase fashion with a kinocilium in the middle (Figures 2D, 3B), while other bundles displayed no clear sign of a staircase pattern (Figures 2C–F). Although the stereocilia of HCLCs appeared to be thinner (Figure 2C), the ultrastructure of the HCLC stereocilia resembled that of hair cells. We determined whether newly emerged stereocilia bundles of HCLCs are functional by directly measuring MET currents in response to bundle displacement (Figure 5). We demonstrated that approximately 75% of HCLCs measured were able to transduce mechanical stimulus, suggestive of the presence of functional MET apparatus in the bundles. Formation of functional bundles in HCLCs suggests that inhibition of Notch signaling is sufficient for morphogenesis of stereocilia bundles and MET apparatus.

HCs contain structural and functional specializations in the basolateral membrane, which are responsible for electrical and mechanical activities. Using TEM and electrophysiological techniques, we determined whether HCLCs developed morphological and electrophysical features that resembled mature hair cells. As shown in Figure 3A, an HCLC derived from a pillar cell maintained its morphological shape of the pillar cells despite the stereocilia bundle on the apical surface. TEM examination showed that the basolateral membrane of HCLCs in the OHC region lacked the subsurface cisternae that are normally present in OHCs. Voltage-clamp recordings further showed that the magnitude and kinetics of outward K^+^ current seen in the HCLCs measured did not resemble those of OHCs or IHCs. None of the HCLCs measured showed NLC. Taken together, these results suggest that the HCLCs examined lacked basolateral membrane specializations of hair cells (e.g., OHCs). It also implies that inhibition of Notch signaling is insufficient for the generation of mature hair cells. We noted that a recent study showed Lgr5-positive supporting cells were converted to prestin-expressing HCLCs after Notch signaling was blocked by application of a γ-secretase inhibitor (LY411575) in tissue culture (Bramhall et al., 2014). Their finding is difficult to reconcile with the present study and some other studies (Kelly et al., 2012; Liu et al., 2012). We would like to point out that lack of hair cell basolateral membrane specializations in HCLCs is unlikely due to the culture environment. Several studies have shown that the development of hair cells in the culture condition follows a similar time course as *in vivo* (Sobkowicz et al., 1975; He and Dallos, 1997; He, 1997; Rüsch et al., 1998; He et al., 2001; Waguespack et al., 2007; Jia et al., 2009). The lack of prestin and subsurface cisternae is also unlikely due to insufficient amount of time for the development of these properties since these properties were examined at the stage when these properties should have been developed—the expression of prestin and appearance of subsurface cisternae occur at P3 and P7 *in vivo* (Souter et al., 1997; Oliver and Fakler, 1999) and *in vitro* (He, 1997; He and Dallos, 1997). We should caution that our observation may only reflect the role of Notch signaling at the time when supporting cells have already acquired their cell fate but still retained some plasticity for conversion. The timing of Notch inhibition and the availability of other genes and regulatory pathways at different developmental stages may explain some differences seen between mutant mice and *in vitro* models. In most mutant mouse models where Notch receptors or ligands are altered during embryotic stage, ectopic hair cells appeared to have the same phenotype as endogenous hair cells (Lanford et al., 1999; Zine et al., 2000). Similar discrepancy has also been observed when *Atoh1* was forced to be over-expressed during embryos vs. after birth. Forced *Atoh1* over-expression at E17 led to supernumerary hair cells with morphological and functional features identical to endogenous hair cells (Gubbels et al., 2008). In contrast, over-expression of *Atoh1* *in vivo* after birth resulted in generation of immature hair cells (Liu et al., 2014).

While formation of stereocilia bundles in supporting cells after inhibition of Notch signaling has been demonstrated by numerous studies (Zine et al., 2000; Yamamoto et al., 2006; Pan et al., 2010; Zhao et al., 2011; Bramhall et al., 2014; Basch et al., 2016; Maass et al., 2016), we showed for the first time that inhibition of Notch signaling led to supernumerary stereocilia in existing hair cells and exuberant growth of microvilli in hair cells and supporting cells in the organ of Corti. This has not been reported before, mainly because few studies have used SEM to examine stereocilia morphology of HCLCs. Although Zhao et al. (2011) used SEM to examine stereocilia bundles in DAPT-treated cultures and observed up to six rows of stereocilia in the organ of Corti, the magnification and orientation of their images were unable to examine individual stereocilia on ectopic hair cells. The supernumerary stereocilia are unlikely due to fusion of cuticular plates of adjacent hair cells as the superfluous stereocilia were clearly due to elongation of microvilli (Figure 4C). Presence of a superfluous small bundle next to the existing bundle resembled stereocilia repair in non-mammalian hair cells when a damaged bundle and a small emerging bundle co-existed (Gale et al., 2002). The fact that altering Notch activity was able to impact stereocilia formation and stability (i.e., the number of stereocilia) in morphologically differentiated supporting cells and hair cells seems to suggest that Notch signaling plays an important role in regulating stereocilia formation and stability.

## Author Contributions

DZZH conceived and designed the experiments. YL, SJ, HL, TT, WG, SY, KWB and DZZH performed the experiments, analyzed the data and generated figures. DZZH and YL wrote the manuscript. All authors read the final version of the manuscript.

## Conflict of Interest Statement

The authors declare that the research was conducted in the absence of any commercial or financial relationships that could be construed as a potential conflict of interest.

## Footnotes

^1^www.SciSoftCo.com
